# Circadian phenotype impacts the brain’s resting-state functional connectivity, attentional performance, and sleepiness

**DOI:** 10.1093/sleep/zsz033

**Published:** 2019-02-15

**Authors:** Elise R Facer-Childs, Brunno M Campos, Benita Middleton, Debra J Skene, Andrew P Bagshaw

**Affiliations:** 1 School of Biosciences, University of Birmingham, Birmingham, UK; 2 Centre for Human Brain Health, University of Birmingham, Birmingham, UK; 3 School of Psychology, University of Birmingham, Birmingham, UK; 4 School of Medical Sciences, University of Campinas, Campinas - SP, Brazil; 5 Faculty of Health and Medical Sciences, University of Surrey, Guildford, UK

**Keywords:** resting-state functional magnetic resonance imaging (fMRI), circadian phenotype, sleep, default mode network, attentional performance, sleepiness, circadian rhythms

## Abstract

**Introduction:**

Functional connectivity (FC) of the human brain’s intrinsically connected networks underpins cognitive functioning and disruptions of FC are associated with sleep and neurological disorders. However, there is limited research on the impact of circadian phenotype and time of day on FC.

**Study Objectives:**

The aim of this study was to investigate resting-state FC of the default mode network (DMN) in Early and Late circadian phenotypes over a socially constrained day.

**Methods:**

Thirty-eight healthy individuals (14 male, 22.7 ± 4.2 years) categorized as Early (*n* = 16) or Late (*n* = 22) using the Munich ChronoType Questionnaire took part. Following a 2-week baseline of actigraphy coupled with saliva samples for melatonin and cortisol rhythms, participants underwent testing at 14:00 hours, 20:00 hours, and 08:00 hours the following morning. Testing consisted of resting-state functional magnetic resonance imaging (fMRI), a structural T1 scan, attentional cognitive performance tasks, and self-reported daytime sleepiness. Seed-based FC analysis from the medial prefrontal and posterior cingulate cortices of the DMN was performed, compared between groups and linked with behavioral data.

**Results:**

Fundamental differences in the DMN were observed between Early and Late circadian phenotypes. Resting-state FC of the DMN predicted individual differences in attention and subjective ratings of sleepiness.

**Conclusion:**

Differences in FC of the DMN may underlie the compromised attentional performance and increased sleepiness commonly associated with Late types when they conform to a societally constrained day that does not match their intrinsic circadian phenotype.

**Statement of Significance**

Misalignment between an individual’s biological timing and behavior (e.g. as a result of shift work or jet lag) has adverse impacts on brain function, performance, and health. We found that people with a late sleep-wake preference, often called “night owls,” have significantly lower functional connectivity in the brain’s “default mode network,” which is involved in maintenance of consciousness and a range of cognitive functions. Importantly, these differences at rest were predictive of poorer attentional performance (slower reaction time) and increased subjective sleepiness. This may represent an intrinsic neuronal mechanism, which leads to “night owls” being comprised during a normal working day. Future work needs to account for these differences while targeting sleep/circadian biology could aid in improving health and performance.

## Introduction

It is estimated that nearly 70 million individuals in the United States alone suffer from some sort of disturbance to the sleep/wake axis which impedes normal functioning and has potentially damaging effects on health and well-being [1]. Societal demands are often in conflict with an individual’s endogenous biological rhythms, leading to adverse impacts on mental and physical health as well as performance. An extreme example of this is shift work, whereby misalignment between an externally imposed work/rest schedule and internal circadian timing can lead to cognitive deficits [2], poorer mental health [3], increased health risks including cancer [4] and a compromised immune system [5].

However, misalignment does not have to be driven by unusual work schedules. By definition, the important issue is that one’s internal temporal organization (i.e. circadian phenotype) and external schedule are in conflict. In particular, a standard working day of 09:00–17:00 hours may be detrimental for an individual whose biological preference is for a late sleep-wake cycle. Compounding the problem, misalignment can also be associated with a cumulative sleep debt, as sleep is curtailed because of late sleep onset, with a similar type and range of adverse outcomes [6]. This aspect of misalignment is much less understood than night shift work, but potentially of greater importance given that, according to estimates from the Office of National Statistics, 12% of the population work night shifts, whereas around 50% have a late preference favoring a wake-up time later than 08:18 hours [7]. Therefore, there is a critical need to increase our understanding of these issues in order to minimize health risks in society and maximize productivity.

It is well established that there are individual differences in circadian timing, that is, diurnal preference [8] and chronotype [7]. At the extreme end of the continuum, these different groups of individuals can be identified as “larks” or “owls” (referred to here as Early [ECP] and Late [LCP] circadian phenotypes based on objective actigraphy and circadian phase markers). Compared to LCPs, ECPs have less disrupted sleep [9], make healthier food choices [10], thereby minimizing risks of obesity and diabetes [11], and reach higher standards in the sports world [12]. Conversely, LCPs have been linked to greater daytime sleepiness [13], increased alcohol consumption and substance abuse [14], decreased psychological well-being through higher rates of depression [15], sleep disorders [16], negative health outcomes [17], and have even been linked to higher mortality rates [18]. Constant desynchronization of their internal circadian rhythms through trying to “fit in” to external societal time, for example, work/school schedules has been suggested as the root cause of these adverse impact on LCPs. This mismatch of biological and social time has been called “social jetlag” [19].

The consequences of sleep and circadian disruption on health and cognitive performance are well established. The application of functional magnetic resonance imaging (fMRI) in this area is still relatively sparse and much of the literature surrounding the relationship between brain function and attention has been focused on task-based fMRI. However, optimal cognitive performance and good mental health rely upon the appropriate coordination of activity between distributed intrinsic functional neuronal networks (often referred to as intrinsically connected networks, ICNs). One ICN, the default mode network (DMN), is particularly affected by sleep onset [20], sleep deprivation [21], variations in habitual sleep patterns across individuals [22], and exhibits diurnal variation in its functional connectivity (FC) [23]. The DMN is most active in the absence of external cognitive demand [24], and has been associated with functions as diverse as self-referential processing [25] maintaining consciousness [26], regulating cognition [27], attention [28], and working memory [29]. It is also modified in a range of psychiatric and neurological disorders [30], including Alzheimer’s disease [31] and depression [32].

Resting-state fMRI provides a complementary approach to task-based fMRI, with the efficiency and integrity of ICNs been linked to intellectual performance [33] and greater intelligence [34], marking the importance that testing resting-state FC (rs-FC) could play in predicting measures of cognitive function. Only a handful of studies have explored the link between FC, sleep, circadian phenotype and cognitive performance [35, 36]. However, these investigations used task-based fMRI and controlled for the effect of circadian phenotype by scheduling testing based on internal biological time, for example, every 4 hours starting 1.5 hours after waking, preventing the exploration of the effect of circadian phenotype in real-life throughout a typical societally constrained day.

In summary, neuroimaging is increasingly used as a technique in sleep research, but inter-subject variability, for example, circadian phenotype brings another level of complexity that is rarely accounted for, despite emerging research showing diurnal variation in brain function [23, 37]. Given that the DMN is evidently vital to basic maintenance of consciousness, affected by sleep alterations, and plays a role in cognitive functioning, it was used as the network of interest in the present study to examine the impact of circadian phenotype on resting-state brain function during the course of a typical societally constrained day (08:00 hours to 20:00 hours). Both anterior (medial prefrontal cortex, mPFC) and posterior (posterior cingulate cortex, PCC) regions of the DMN were used as seed regions to gather information about the functional integrity of the DMN at rest, and these data linked to attentional performance and sleepiness outside of the MRI scanner. We hypothesized that LCPs would show disrupted FC compared to ECPs, and that FC differences would be correlated with behavior.

## Methods

### Participants

The study was approved by the University of Birmingham Research Ethics Committee. Individuals (*n* = 204) from the University of Birmingham and surrounding community completed the Munich Chronotype Questionnaire (MCTQ [38]) and were screened for any contraindications to inclusion in the study based on medical history and magnetic resonance safety. Exclusion criteria were (1) no prior or current diagnoses of sleep, neurological or psychiatric disorders; (2) taking medications that affect sleep or melatonin/cortisol rhythms; and (3) intermediate chronotype indicated by corrected mid-sleep times on free days (MSF_sc_) from the MCTQ.

A total of 38 healthy individuals (14 male, 22.7 ± 4.2 years) who were categorized as “Early” (*n* = 16, age 24.7 ± 4.6 years, nine female, MSF_sc_ 02:24 ± 00:10 hours) or “Late” (*n* = 22, age 21.3 ± 3.3 years, 15 female, MSF_sc_ 06:52 ± 00:17 hours) chronotypes and who also passed all inclusion criteria were invited to take part in the main study. Participants gave written informed consent before involvement and all details provided were given on a voluntary basis. After completing questionnaires, physiological sampling and between 13–16 days of actigraphy in their home environment (details below), participants attended the Birmingham University Imaging Centre for testing sessions at 14:00 hours, 20:00 hours, and 08:00 hours (GMT) the following morning. Individuals went home in between testing sessions. Testing sessions were conducted in a specific order (14:00 hours, 20:00 hours, and 08:00 hours) to prevent the 14:00 hours and 20:00 hours sessions being affected by sleep deprivation. This design allowed all individuals to wake-up naturally for the 14:00 hours and 20:00 hours. Summary details of participants’ data can be found in Table 1. At each testing session, participants underwent a resting-state fMRI and T1 scan followed by cognitive testing (psychomotor vigilance task [PVT] and Stroop task) and subjective sleepiness ratings (details below). As part of the cognitive testing that was completed at each session, a questionnaire was developed and administered to gather details about what was occurring between sessions when participants left the laboratory. In an attempt to partially control for external variables and confirm no differences between the groups, information gathered included hours since (1) food intake; (2) caffeine consumption; (3) exercise; (4) exposure to natural light; and (5) exposure to indoor light (Table 1).

**Table 1.** Summary of demographic, actigraphic and physiological variables for ECPs and LCPs

Variable measured (mean ± SEM)	ECPs	LCPs	Significance
Demographic variables			
Sample size	*N* = 16	*N* = 22	n/a
Number of scans/testing sessions	*N* = 48	*N* = 66	n/a
Percentage of males/females (%)	*M* = 43.8	*M* = 31.8	ns^c^
	*F* = 56.3	*F* = 68.2	ns^c^
Age (years) (mean ± *SD*)	24.7 ± 4.0	21.2 ± 3.3	*p* = 0.028^a^
Height (cm)	171.3 ± 2.0	171.1 ± 2.4	ns^a^
Weight (kg)	66.4 ± 2.8	67.1 ± 2.1	ns^a^
MCTQ score (hours:minutes)	02:24 ± 00:10	06:52 ± 00:17	*p* < 0.0001^a^
Actigraphic variables			
Sleep onset (hours:minutes)	22:57 ± 00:10	02:27 ± 00:19	*p* < 0.0001^a^
Wake-up time (hours:minutes)	06:33 ± 0.10	10:13 ± 00:18	*p* < 0.0001^a^
Sleep duration (hours)	7.59 ± 0.18	7.70 ± 0.14	ns^a^
Sleep efficiency (%)	79.29 ± 1.96	77.23 ± 1.14	ns^a^
Sleep onset latency (hours:minutes)	00:25 ± 00:06	00:25 ± 00:03	ns^b^
Physiological variables			
Phase angle (hours:minutes)	02:28 ± 00:16	02:34 ± 00:18	ns^a^
Dim light melatonin onset (hours:minutes)	20:27 ± 00:16	23:55 ± 00:26	*p* < 0.0001^a^
Cortisol peak time (hours:minutes)	07:04 ± 00:16	11:13 ± 00:23	*p* < 0.0001^a^
External variables (between sessions)			
Hours since last meal (hours)	3.58 ± 0.55	5.07 ± 0.58	ns^b^
Hours since caffeine (hours)	8.47 ± 0.67	7.85 ± 0.82	ns^b^
Hours since exercise (hours)	6.78 ± 0.74	7.44 ± 0.74	ns^b^
Hours since natural light exposure (hours)	5.87 ± 0.80	3.51 ± 0.58	ns^b^
Hours since indoor light exposure (hours)	1.88 ± 0.38	3.32 ± 0.51	ns^b^

Values are shown as mean ± SEM unless specified. Significance is shown with ^a^parametric tests, ^b^nonparametric tests or ^c^Fisher’s exact test. Phase angle is calculated by the interval time between dim light melatonin onset and sleep onset.

### Sleep analysis

Actigraphs (Actiwatch Light, AWLs, 2006, Cambridge Neurotechnology Ltd) were worn on participants’ nondominant wrist to gather activity and light exposure data (1–32 000 lux) for 13–16 days prior to testing sessions. This allowed sleep and activity patterns to be monitored continuously in the home environment. Data were acquired in 1-minute epochs (medium sensitivity setting), confirmed with daily sleep diaries, and analyzed using Sleep Analysis 7 Software (version 7.23, Cambridge Neurotechnology Ltd). Throughout this period participants were following preferred routines and were not confined to particular schedules.

### Physiological data

Saliva samples were provided during one morning and one evening the week of testing by spitting into pre-labeled polypropylene collection tubes (7-ml plastic bijou) following strict standardized protocols. Participants were trained in how to take the saliva samples in their home environment during their initial set up visit and the protocol instructions were discussed to ensure participants understood what was required. In addition, a sample collection record sheet was attached to both morning and evening protocols to ensure that the exact times samples were taken could be recorded. During the sampling periods, participants were asked to abstain from caffeinated drinks, alcoholic drinks or any drinks containing artificial coloring. They were also asked to refrain from cleaning their teeth, chewing gum or going to the bathroom at least 15 minutes before each sample. Evening samples were collected from a seated position while in dim lighting conditions (no overhead lights, no electronic screens, and curtains closed) every 30 minutes from 3 hours prior to individual habitual bedtime until 1 hour after. Morning samples were collected on awakening, every 15 minutes for the first hour and every 30 minutes for the following 2 hours. All samples were anonymized. Radioimmunoassays (RIA) of melatonin and cortisol were performed (Stockgrand Ltd, University of Surrey) using an Iodine-125 radioactive labeled tracer and solid phase separation [39]. Assays were run with quality controls (QCs) before and after samples. These QC values were then averaged to give one value per assay to calculate inter-assay coefficients of variation (CV %). The limit of detection for the melatonin assay was 0.72 ± 0.08 pg/ml and CVs were 9.4% at 44.4 pg/ml, 9.9% at 20.1 pg/ml and 12.2% at 9.0 pg/ml (*n* = 13 at each concentration). The limit of detection for the cortisol assay was 0.45 ± 0.06 nmol/L and inter-assay CVs were 8.3% at 48.0 nmol/L, 6.1% at 15.9 nmol/L, and 9.8% at 3.0 nmol/L (*n* = 15 at each concentration).

Individual dim light melatonin onset (DLMO) values were calculated using the mean of the individual baseline concentration values plus two *SD*s of the mean. Due to intra-subject variability in melatonin concentrations these calculations were performed relative to each individual. This concentration was used to calculate the timing of melatonin onset through a linear response function. The peak time of the cortisol awakening response was calculated as the time of highest cortisol concentration recorded. All results were calculated based on individual sample timings taken from sample collection record sheets. Due to insufficient or contaminated samples, DLMO values were unable to be calculated for two ECPs and four LCPs.

### Neuroimaging acquisition

Imaging data were acquired using a Philips Achieva 3T MRI scanner with a 32-channel head coil. Whole brain coverage gradient echo-planar imaging data were acquired parallel to the AC-PC line with the following parameters: 15 minutes, 450 volumes, TR = 2000 ms, TE = 35 ms, flip angle = 80°, 3 × 3 × 4 mm voxels, 32 slices, no gap, matrix = 80 × 80 × 32. Standard high-resolution 3D anatomical T1-weighted scans (sagittal acquisition, TR = 8.4 ms, TE = 3.8 ms, flip angle = 8°, 1 mm isotropic voxel, matrix = 288 × 288 × 175) were also collected to facilitate co-registration. Respiratory and cardiac fluctuations were recorded with the pulse oximeter and pneumatic belt provided by the scanner manufacturer. A camera was placed in the scanner during each session to monitor participants’ eyes, confirm they remained open and that sleep had not been initiated. If eye closure exceeded 15 seconds, which is half a 30-second epoch according to the standard sleep staging approach [40], the scan was re-started. This occurred in one scan for one participant. Standard Birmingham University Imaging Centre operating procedures were followed for the MRI safety screening and during the scanning sessions, and participants were not asked to perform any task.

### Neuroimaging preprocessing

FMRI preprocessing and analysis was performed using UF^2^C [41], PhysIO [42], and SPM12 [43] toolboxes implemented in MATLAB (MathWorks, United States). Preprocessing was carried out in UF^2^C using standardized methodologies implemented in SPM12. Data were reorientated to the anterior commissure as origin, motion corrected using rigid body transformations (three translational and three rotational planes), spatially normalized (MNI-152 template space), spatially smoothed with a 6-mm Gaussian kernel and detrended (temporal linear trends removal). Physiological noise corrections (RETROICOR for a third order cardiac, fourth order respiratory, and first order interaction Fourier expansion of cardiac and respiratory phase, heart rate variability and respiratory volume per time) were modeled using the PhysIO toolbox. This resulted in 18 nuisance regressors which were added to preprocessing routines in UF^2^C, along with average signals for white matter (WM) and cerebrospinal fluid (CSF) and six movement (three translational and three rotational) regressors. High-pass (>0.008 Hz) and low-pass (<0.1 Hz) temporal filtering was applied to remove confounding physiological frequencies. Framewise displacement (FD) and derivative variance (DVARs) were calculated [44, 45], and any scan with an average FD value above 0.5 mm was excluded. This resulted in one scan (ECP, 14:00 hours) being removed from further analysis. Head movement (translational, rotational, FD, and DVARS) did not differ significantly between the groups or between times of the day.

### Neuroimaging analysis

A seed-based FC approach was used to analyze the data using predefined seeds for the frontal (mPFC) and posterior (PCC) regions of the DMN [46]. Pearson correlation maps were then converted to *z*-score maps using Fisher’s Transformation. Using the general linear model (GLM) implemented in SPM12, second-level group analyses were performed using a flexible factorial design. The second-level analyses were performed using a voxel-level threshold FWE corrected at *p* < 0.05. A subsequent extent threshold (FWE corrected at *p* < 0.05) was used to concentrate on the significant results at the cluster level. Subject, group and time of day were added as factors and the model was set up for the main effect of group (ECPs and LCPs), the main effect of time of day (morning; 08:00 hours, afternoon; 14:00 hours, and evening: 20:00 hours) as well as the interaction of group and time of day. All subject variability including age and gender were accounted for as covariates by adding subject as a factor. Descriptions of significant findings from the mPFC seed (voxel-level threshold FWE corrected at *p* < 0.05, with a subsequent extent threshold of 100 voxels) and PCC seed (voxel-level threshold FWE corrected at *p* < 0.05, with a subsequent extent threshold of 150 voxels), are presented as total voxels, peak *t* score and peak MNI centroid cluster coordinates [x y z]. Extent thresholds were selected as a fifth of the biggest cluster. All significant areas were transformed in a binary mask and the *z*-scored values from the correlation map within this mask were averaged generating a single value representing average rs-FC across all significant clusters per participant for each scan. These values were used to explore the predictive effects of rs-FC on attention and daytime sleepiness using generalized estimating equations (details given in Statistical Analysis section).

### Attentional performance and sleepiness

Following the scan, participants were immediately taken to a testing room where a 2-minute PVT [47] and a Stroop Colour-Word Task [48] were completed. A visual version of the Stroop test was used which consisted of 60 trials with equal proportion of congruent and incongruent stimuli (30 of each). Presentation time was not fixed, that is, stimuli were visible until response. Reaction time values were used from the PVT and the Stroop task (averaged correct congruent and incongruent trials) as indices of attentional performance. Incompletion of the Stroop test resulted in one participant’s results being excluded for further analysis. Daytime sleepiness, measured using the Karolinska Sleepiness Scale (KSS) [49], was completed before the cognitive tests were performed.

### Statistical analysis

Statistical comparisons of behavioral data were performed in GraphPad Prism (version 7, La Jolla, CA) and SPSS (IBM SPSS Statistics, version 24, Chicago, IL) using two-sided unpaired *t*-tests, Mann-Whitney U tests, Fisher’s exact test, and linear regression after testing for equality of means with Levene’s test. All *p*-values were FDR corrected to control for multiple comparisons [50]. Diurnal variations in performance and sleepiness variables were plotted using second-order regression curves and analyzed using two-way analysis of variance (ANOVA) for repeated measures with post hoc multiple comparison tests. Nonparametric tests were implemented where data did not follow a normal distribution.

To explore the predictive effects of rs-FC on performance variables and daytime sleepiness an extension of the generalized linear model (generalized estimating equations, GEEs) were used in SPSS. GEEs account for repeated measures and within-subject variability and do not assume normal distributions or linear relationships. GEEs are often used in studies with time of day data to model the average effect, and have been used in sleep and circadian research to model the relationship between insomnia, depression, and chronotype [16] as well as in studies on sleep durations [51, 52] and circadian patterns in epilepsy [53]. Data used in GEE analyses were *z*-scored average rs-FC values across all clusters for each participant, individual reaction times (PVT and Stroop) and KSS score. A scale linear response GEE with identity link function for scale data was used to model the independent effects of rs-FC on attentional performance. A negative binomial GEE with log link function for count data was used to model the effects of rs-FC on sleepiness. Both models were designed adding Subject ID as a subject variable, and circadian phenotype (ECP/LCP) and time of day (08:00 hours, 14:00 hours, and 20:00 hours) as within-subject variables. Time of day was also added as a fixed factor. When interaction terms were not significant they were removed from the model and the analysis re-run. Corrected quasi-likelihood under independence model criterion (QICC) values were used to choose the best fit for models.

Significance levels are displayed as not significant (ns), *p* < 0.05 (*), *p* < 0.01 (**), *p* < 0.001 (***), and *p* < 0.0001 (****). Exact *p* values are given apart from when significance is identified as less than 0.0001, in which case *p* < 0.0001 is reported. Results are shown using the mean ± standard error of the mean (SEM) unless specified otherwise.

## Results

### Circadian phenotyping

Individuals were initially categorized into Early (*n* = 16) and Late (*n* = 22) chronotypes using MSF_sc_, calculated using the MCTQ [38]. These groups were confirmed as ECPs and LCPs by analysis of biological circadian phase markers, namely DLMO and time of peak morning concentration of the cortisol awakening response, in addition to sleep start and wake-up times calculated from actigraphy analysis. All parameters were significantly different between the groups, occurring approximately 3.5–4.5 hours earlier in ECPs than LCPs (Table 1). MSF_sc_ was ~4 hours earlier in ECPs compared to LCPs (*t*(36) = 12.2, *p* < 0.0001). DLMO and peak time of morning cortisol also differed significantly by ~3.5 hours and ~4 hours, respectively (*t*(30) = 6.8, *p* < 0.0001 and *t*(36) = 8.0, *p* < 0.0001). These results were consistent with sleep onset and wake-up times calculated from actigraphy data, with a difference between the groups of ~3.5 hours (*t*(34) = 8.9, *p* < 0.0001 and *t*(34) = 9.9, *p* < 0.0001).

**Figure 1.**
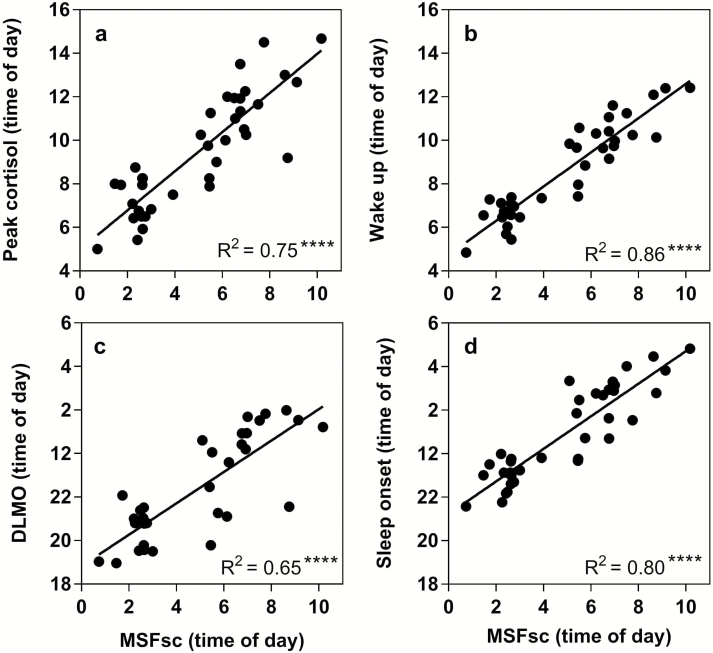
Linear relationships between corrected mid-sleep on free days (MSF_sc_) and biological phase markers to validate circadian phenotyping. (a) Dim light melatonin onset (DLMO), (b) Sleep onset, (c) Time of peak cortisol concentration, (d) Wake-up time. MSF_sc_ is displayed as time of day (hours) on the x-axis. Statistical analysis was carried out using linear regression analysis. Significance (****p < 0.0001) and *R*^2^ values are shown in the bottom right corner.

Each of these parameters was significantly correlated with MSF_sc_ (Figure 1). Significant linear regressions were found between MSF_sc_ and DLMO (*R*^2^ = 0.65, *p* < 0.0001), peak time of cortisol awakening response (*R*^2^ = 0.75, *p* < 0.0001), sleep onset (*R*^2^ = 0.80, *p* < 0.0001) and wake-up time (*R*^2^ = 0.86, *p* < 0.0001). All other actigraphic parameters were not significantly different between ECPs and LCPs (Table 1). As all participants in this study were following their preferred schedules for the duration of the experiment, these findings confirmed that neither group were acutely sleep deprived during the baseline period. However, in order to rule out a baseline sleep debt effect, additional analyses were run to examine the relationships between sleep efficiency and rs-FC. No significant correlations were found. These results support the classification into circadian phenotypes and demonstrate that these two groups are behaviorally and physiologically different in sleep timings and circadian phase but not in other sleep parameters.

### Resting-state functional connectivity in circadian phenotypes

**Figure 2.**
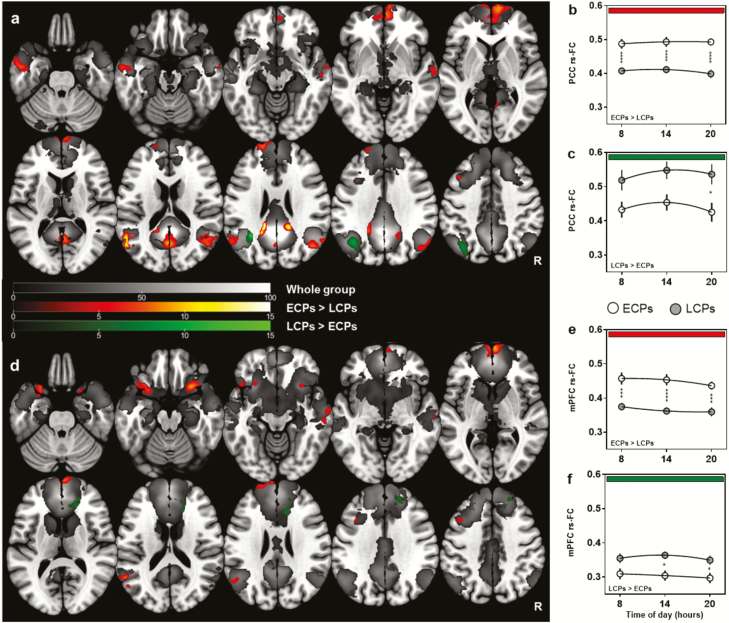
rs-FC of the Default Mode Network between ECP and LCP. *z*-transformed connectivity maps show significant clusters (FWE-corrected *p* < 0.05 at voxel level and subsequent cluster level) and *t*-score scales for each contrast are shown in the center. Overall results from each seed are shown in a/d with results from each time point (hours) represented in b/c and e/f. (a) Summary results from the PCC seed with diurnal variations between circadian phenotype groups plotted in (b) and (c). (d) Summary results from mPFC seed with diurnal variations between circadian phenotype groups plotted in (e) and (f). Significant regions at the whole group level are represented in grayscale. Regions higher in ECPs (ECPs > LCPs) are shown in red and regions higher in LCPs (LCPs > ECPs) in green. Statistical analysis for (a) and (d) was carried out using a flexible factorial model in SPM12. Two-way ANOVA was used to analyze group and time of day differences in (b), (c), (e), and (f). *p < 0.05, ***p < 0.001, ****p < 0.0001.

Whole group analyses showed a clear DMN from both seeds, with significant FC (FWE corrected *p* < 0.05) observed between all major components of the DMN including the PCC/precuneus, mPFC, bilateral angular and temporal gyri, and cerebellum (Figure 2, grayscale underlay).

The flexible factorial model showed clear significant differences between circadian phenotype groups but no significant main effect of time of day (Figure 2). ECPs had significantly increased FC compared to LCPs at all times of day in 15 of the total 18 supra-threshold clusters identified from both seeds (FWE corrected at *p* < 0.05). When seeding in the PCC, there was significantly higher FC for ECPs from PCC to the precuneus, bilateral angular gyri, left medial temporal lobe, and cingulate gyrus. The largest cluster was found in the mPFC, along with two clusters in the left medial frontal and superior frontal lobe (Table 2; Figure 2, a and b). When seeding in the mPFC there was, again, significantly higher FC in ECPs from the seed to seven individual clusters including: within the mPFC, bilateral insula, left medial frontal lobe, left angular gyrus, left superior frontal gyrus, and right medial temporal lobe (Table 2; Figure 2, d and e).

**Table 2.** Summary of significant brain regions (FWE, *p* < 0.05) between ECPs and LCPs when seeding in the PCC and mPFC

Region	Contrast	Seed region	Cluster size (voxels)	MNI centroid coordinates [x y z]	Maximum *t*-score
mPFC	ECPs > LCPs	PCC	789	[−2 72 12]	13.71
Right angular gyrus	ECPs > LCPs	PCC	481	[46 −68 26]	8.14
Precuneus	ECPs > LCPs	PCC	431	[0 −64 18]	9.73
Left angular gyrus	ECPs > LCPs	PCC	257	[−54 −62 18]	14.75
Left medial temporal lobe	ECPs > LCPs	PCC	237	[−58 −6 −24]	7.94
Left superior frontal gyrus	ECPs > LCPs	PCC	212	[−18 60 26]	7.91
Left medial frontal lobe	ECPs > LCPs	PCC	173	[−46 16 56]	8.71
Cingulate gyrus	ECPs > LCPs	PCC	150	[−16 −42 26]	18.90
Left angular gyrus	LCPs > ECPs	PCC	428	[−32 −54 26]	16.29
mPFC	ECPs > LCPs	mPFC	384	[2 70 6]	10.99
Left anterior insula	ECPs > LCPs	mPFC	378	[−26 14 −24]	8.87
Right anterior insula	ECPs > LCPs	mPFC	241	[26 18 −20]	9.36
Left medial frontal lobe	ECPs > LCPs	mPFC	160	[−44 16 56]	9.62
Left angular gyrus	ECPs > LCPs	mPFC	134	[−56 −58 18]	10.19
Left superior frontal gyrus	ECPs > LCPs	mPFC	111	[−4 68 28]	8.96
Right medial temporal lobe	ECPs > LCPs	mPFC	108	[68 −12 −8]	6.15
Anterior cingulate	LCPs > ECPs	mPFC	233	[22 44 10]	7.20
Right superior frontal gyrus	LCPs > ECPs	mPFC	161	[22 42 52]	6.55

In comparison, LCPs had higher FC to three of the 18 identified clusters that survived FWE correction at *p* < 0.05. When seeding in the mPFC, clusters were found in the anterior cingulate cortex and right superior frontal gyrus, while seeding in the PCC identified a cluster in the left angular gyrus (Table 2; Figure 2, c and f).

### Attentional performance and sleepiness

**Figure 3.**
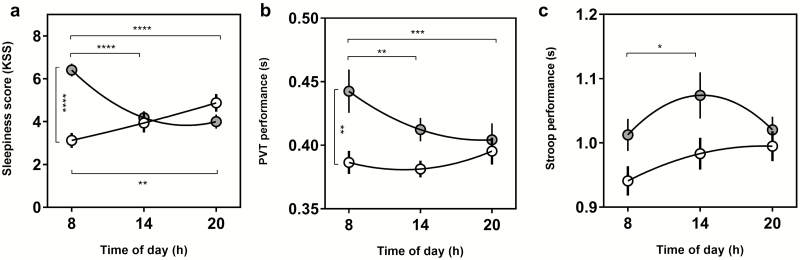
Nonlinear regression curves to show diurnal variations in sleepiness, PVT and Stroop performance. (a) Subjective sleepiness score measured with the Karolinska Sleepiness Scale. (b) PVT performance (reaction time in seconds), (c) Stroop performance (reaction time in seconds) for Early circadian phenotypes (white) and Late circadian phenotypes (gray). Clock time of test (hours) is shown on the *x* axis for each parameter. Statistical analysis was carried out using two-way ANOVA. Post-hoc multiple comparison tests were run to determine group and time of day effects. *p < 0.05, **p < 0.01, ***p < 0.001, ****p < 0.0001.

A significant interaction between circadian phenotype and time of day was found for PVT performance (*F*(2, 72) = 4.9, *p* = 0.01) but not Stroop performance (*F*(2, 70) = 1.6, *p* = 0.22). The main effect of time of day was significant for both PVT (*F*(2, 72) = 3.2, *p* = 0.048) and Stroop performance (*F*(2, 70) = 3.8, *p* = 0.028) as well as the main effect of circadian phenotype for PVT (*F*(1, 36) = 4.4, *p* = 0.044) but not Stroop (*F*(1, 35) = 3.7, *p* = 0.063) (Figure 3, b and c). Post-hoc tests revealed that the source of group effect for PVT was the 08:00 hours testing session, where LCPs’ performance was significantly worse than ECPs (*p* = 0.0058). Significant diurnal variations were found for LCPs but not ECPs in both PVT and Stroop performance, showing that the source of time of day effects were driven LCPs. LCPs morning PVT performance was significantly worse compared to the afternoon and evening (*p* = 0.0079 and *p* = 0.0006). LCPs morning Stroop performance was significantly better in the afternoon compared to morning (*p* = 0.035).

For the KSS, there was a significant interaction between time of day and circadian phenotype (*F*(2,72) = 18.1, *p* < 0.0001), as well as a significant main effect of circadian phenotype (*F*(1,36) = 9.2, *p* = 0.0044) but not time of day (*F*(2,72) = 2.0, *p* = 0.15). Group effects were driven by LCPs being significantly sleepier at 08:00 hours compared to ECPs (*p* < 0.0001). The interaction effect revealed significant diurnal variations in both groups with opposing relationships. ECPs were significantly more sleepy in the evening (4.9 ± 0.4) compared to the morning (3.1 ± 0.4) (*p* = 0.0054). LCPs showed the inverse relationship being significantly sleepier at 08:00 hours (6.4 ± 0.3), compared to 14:00 hours and 20:00 hours (both *p* < 0.0001) (Figure 3a).

### Predicting attentional performance and sleepiness

**Figure 4.**
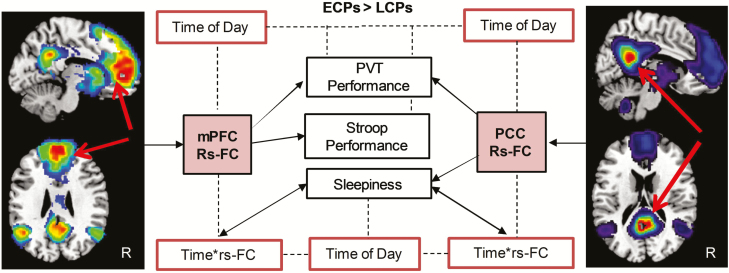
Summary of predictive analysis using rs-FC to predict attentional performance and subjective daytime sleepiness (black boxes). Solid arrows indicate the predictive effects of rs-FC on attentional performance (PVT and Stroop task) and sleepiness variables for models using data from seeds in the mPFC and PCC. Dotted lines and red boxes indicate where time of day or the interaction of time of day and rs-FC was also found to be a significant factor.

rs-FC could independently predict performance variables (Figure 4). Using FC values from regions with higher FC in ECPs than LCPs, GEEs showed that rs-FC of the mPFC could predict PVT (*W* = 14.5, *p* < 0.0001) and Stroop performance (*W* = 9.0, *p* = 0.003). Rs-FC of the PCC (ECPs > LCPs) could also predict PVT performance (*W* = 6.4, *p* = 0.012) but not Stroop performance (*W* = 2.5, *p* = 0.12). Sleepiness score could be predicted by rs-FC of the PCC (*W* = 6.0, *p* = 0.015) but not rs-FC of the mPFC (*W* = 1.5, *p* = 0.22). No significant predictive effects of rs-FC were found for regions higher in LCPs (LCPs > ECPs) for either seed.

Time of day was also a significant independent predictor of performance and sleepiness. Using the mPFC model, time of day could predict PVT (*W* = 9.2, *p* = 0.01) but not Stroop performance (*W* = 5.1, *p* = 0.078). Using the PCC model, time of day was a significant predictor of both PVT and Stroop performance (*W* = 6.3, *p* = 0.042 and *W* = 7.1, *p* = 0.028, respectively). Sleepiness could be independently predicted by time of day (mPFC: *W* = 17.1, *p* < 0.0001 and PCC: *W* = 11.1, *p* = 0.004) as well as by the interaction of rs-FC and time of day for both models (mPFC: *W* = 14.5, *p* = 0.001 and PCC: *W* = 8.7, *p* = 0.013).

In summary, averaged rs-FC of the mPFC from the regions higher in ECPs compared to LCPs predicted better attentional performance, that is, faster reaction times in both PVT and Stroop performance. Similarly, the equivalent measures from the PCC seed could predict better PVT performance and lower daytime sleepiness but not Stroop performance. The interaction of time of day and rs-FC predicted daytime sleepiness for both seeds. Time of day independently predicted attentional performance and sleepiness variables in both models. Averaged rs-FC from regions showing higher FC in LCPs compared to ECPs for both seeds showed no predictive effects on attentional performance or sleepiness, with only time of day predicting PVT and Stroop performance.

## Discussion

According to previous research only around 15% of the population falls into extreme or moderate Early chronotypes (going to sleep between 20:30 and 23:00 hours and waking between 04:30 and 07:00 hours) [7], meaning the majority of the population would not usually fit into the standard working schedule, preferring to go to sleep and wake-up later. Consequently, many individuals, in particular, those with extreme late preferences who can be classified as LCPs, are constantly fighting their innate circadian phenotype and sleep patterns to fit into socio-professional routines.

Here we show, for the first time, fundamental differences in FC of the DMN between ECPs and LCPs during a typical working day (08:00–20:00 hours). Regardless of time of day, ECPs had higher rs-FC than LCPs in the majority of regions identified. Many of the regions identified as having higher rs-FC in ECPs are linked to cognitive function and control, including the right and left anterior insula (rAI and lAI), two main regions which are also featured in the salience network. FC between the mPFC and the rAI has previously been shown to correlate with cumulative habitual sleep duration [22], and with the current data this suggests that mPFC-insula FC during wakefulness could also be sensitive to sleep timings and circadian phenotype. Given that connectivity between similar regions are associated with either sleep duration or timing, these regions could be more broadly related to sleep and highlight the potential importance of inter-network connectivity. Furthermore, rs-FC of these regions was predictive of attentional performance measures, that is, reaction time and subjective sleepiness. While we are not able to identify the causality of the relationships unambiguously within our experimental design, this could suggest that the higher rs-FC of the DMN observed in ECPs over relatively widespread regions mediates improved task performance. It is also important to note that while the interpretation of FC can be partially based on activation studies using task-based fMRI, the relationship between connectivity and activation is not straightforward and remains an active area of research [54, 55].

Interactions between the brainstem arousal systems and ventrolateral preoptic nucleus of the hypothalamus are known to play in determining circadian rhythmicity and sleep-wake cycles. The impact of an underlying biological predisposition (e.g. circadian phenotype) to particular sleep-wake patterns on brain function and subsequently behavior has not previously been demonstrated, but is consistent with previous observations linking FC to behavioral performance [56] and habitual sleep durations [22]. Therefore, an alternative proposal would be that there could be other brain regions, shown here in DMN FC, that contribute to variability between circadian phenotypes. These differences in intrinsic FC have not previously been linked to the known role of the DMN presenting an interesting area for future research.

Of the 18 regions identified as being significantly different in terms of their FC between ECPs and LCPs, the substantial majority (83%) demonstrated higher FC in ECPs. This suggests that an early sleep-wake pattern is generally associated with higher FC from the primary nodes of the DMN. Since the 08:00 hours session required LCPs to wake earlier, these individuals were suffering from acute sleep restriction. As a result, the morning session was expected to show the greatest difference between the groups. PVT performance and sleepiness scores exhibited significant diurnal variations and were significantly lower in LCPs compared to ECPs at 08:00 hours, suggesting that these measures could be sensitive to the curtailment in sleep. However, this result is not reflected in FC, which shows consistent group differences at each time point but no significant diurnal variations. As such, these findings could be due to more intrinsic circadian phenotype traits and not acute sleep restriction. While LCPs tend to be heavily disrupted throughout their lifetimes when enforced to fit conventional societal days, those taking part in the current study were able to follow their own preferred routines throughout the study and had comparable sleep parameters to ECPs (e.g. duration, efficiency) with only sleep timings differing significantly. This would support the notion of LCPs showing adverse effects when persistently following an earlier schedule during the work week, even when trying to compensate on nonworking “free” days [19]. It is likely that a more chronic effect of long-term misalignment, for example, years of having to fit into school and subsequent work schedules, may extend to impact on intrinsic brain properties even when individuals are able to follow their own schedules for a period of 2 weeks. This is consistent with observations of continued cognitive deficits following prolonged shift work, even after the shift work has ceased [57]. Therefore, these findings may be underestimating the differences in FC and performance, which could be exacerbated by acute disruption.

The increasingly sophisticated ability of fMRI to probe and quantify the human brain’s functional architecture opens up new possibilities for understanding the impact of sleep and circadian preferences at the level of the individual. While considerable advances have been made in understanding the cellular and genetic underpinnings of sleep and circadian rhythmicity [58], and behavioral effects have been characterized [7], only recently have the methods been available to study their impacts on the human brain in vivo. These developments are crucial, given the intrinsic importance of understanding human brain function and the commonly-held view that the primary purpose of sleep is for the brain [59]. The use of rs-FC is particularly attractive for this endeavor because of the pervasiveness of the behavioral and cognitive effects of sleep patterns and circadian phenotype, which lend themselves to characterization of intrinsic network function rather than the more limited task responses. More broadly, the approach we have taken provides important information about how intrinsic lifestyle factors and biological phenotypes are reflected in the brain’s default state (DMN), suggesting new avenues for understanding individual differences in behavior.

Our analysis revealed that rs-FC of the DMN can independently predict measures of task performance and subjective daytime sleepiness. This suggests that the higher strength of rs-FC between these regions, the better an individual performs in an attention task and the less sleepy they feel. Since our analysis used seeds within the DMN, one could infer that the functional integrity of connections from key regions of the DMN facilitates attentional performance, and that perturbations of the DMN associated with misalignment are detrimental (caveats regarding causality as discussed above notwithstanding). The DMN is important in maintenance of consciousness and includes cognitive domains sub-served by the frontal cortex [60]. Altered FC of the DMN has been reported in a number of psychiatric disorders, suggesting that disrupted integrity of this network is linked to psychological processes (see [61] for review). Although decreased FC does not always relate to decreased task performance, reduced connectivity from mPFC and PCC regions of the DMN has been proposed to underlie impairments in attentional control, working memory and emotional processing [61]. The majority of this research, investigating both DMN connectivity and activation, has reported decreased FC in disorders such as Alzheimer’s, attention deficit hyperactivity disorder and autism. Conversely, an increase in FC from the subgenual anterior cingulate has been associated with depression [62]. We find that ECPs have higher rs-FC from the majority of significant clusters. However, of the three clusters that we identify as having higher rs-FC in LCPs, one was in the anterior cingulate cortex. Since LCPs are a group who have frequently been linked to higher rates of depression, this result has potentially uncovered an interesting avenue for future work and highlights that interpreting increases/decreases in rs-FC are not always straightforward. Adding to the growing body of research into the consequences of disrupted DMN rs-FC, we now show that circadian and sleep variations can contribute to understanding how the integrity of the DMN at rest could hold a key role in achieving optimal cognitive functioning (shown here using attentional tasks).

Previous research has shown diurnal variations in FC of resting-state networks, suggesting that different ICNs have varying sensitivity to time of day [23, 37]. However, although in the current study diurnal variations were found in attentional performance and sleepiness measures, using a flexible factorial design to account for the complex study design, we found that the effect of circadian phenotype on rs-FC was much more marked than the effect of time of day. This suggests that rs-FC of the DMN is primarily sensitive to stable, trait-like differences between the two groups rather than more dynamic state-like effects. This is consistent with the fact that habitual sleep patterns have been linked with anatomical [63] as well as functional [22] differences, suggesting long-term modifications to brain function can occur as a result of modifications to the underlying structure. However, it is possible that the examination of additional networks beyond the DMN and the use of dynamic FC [64] would identify state-like impacts of circadian misalignment which might be more sensitive to the effects of time of day. It is also important to note that these data were gathered during typical working hours (08:00–20:00 hours) which could have resulted in failure to record time points in which LCPs could have shown higher FC and better attentional performance. However, LCPs are under constant pressure to fight again their endogenously driven circadian rhythms to fit into socio-professional imposed schedules. This could cause them to be in a state of “perpetual chronodisruption” despite being able to follow their preferred schedules for the duration of this study.

There are a number of limitations to this study. Firstly, to be able to investigate how ECPs and LCPs behave during a “normal socially constrained day,” for example, 08:00–20:00 hours, the study was designed using clock time instead of scheduling testing based on internal biological time. Although this design does not allow sleep and circadian influences to be separated, there is an increasing need to carry out “real-world” studies to increase external validity as behavior is impacted by both factors. In addition, we only investigated one ICN, the DMN, and therefore limit the ability to explore more complex whole-brain inter- and intra-network FC. Both the mPFC and PCC regions of the DMN were used as seeds because although the DMN is a coherent network, each of the regions that comprise it also have other functions and potentially have different susceptibility to the impact of circadian phenotype and time of day. Since the DMN is the most widely studied ICN, holds a key role in maintenance of consciousness, is affected by sleep, and disruption of this network has been linked to impaired attentional control, there was a strong rationale to choose it as the network of interest and provides a useful starting point for a relatively unexplored field. Nonetheless, studying the impact of circadian phenotype on other ICNs, as well as other measures of cognition which could be impacted differently, would be an important next step for future work. Similarly, given that ECPs and LCPs differ significantly in their physiology, another important step would be to explore biological and genetic mechanisms behind the observed changes in rs-FC.

The majority of variables were evenly matched between the groups with the exception of sleep timings (onset/offset) and circadian phase markers (DLMO). Sleep efficiency values were relatively low for healthy controls, although sleep durations are in the normal range for this cohort of young adults and additional analysis showed no significant correlations of sleep efficiency and rs-FC. This suggests that there is no baseline sleep debt effect and both groups are not acutely suffering from sleep debt during the course of this study. This allows us to confidently state we have distinct circadian phenotype differences. We did have a slight but significant difference in age between the groups, although not sufficient to account for the differences since studies examining the relationship between FC of the DMN and age demonstrate that FC is stable from young adulthood until 50–60 years [65].

Throughout the duration of the study, participants were following their preferred routines to allow a true indication of the impact of circadian phenotype in the absence of masking effects. However, this is likely to underestimate the practical impact on LCPs of conforming to a societal day, since in reality the LCPs are likely to have an additional burden of sleep debt which will have its own negative effect. In our study, the 08:00 hours session will have caused the LCPs to wake earlier than usual and, therefore, be affected by sleep restriction. Although we are not able to determine the extent of shortening the sleep period before the morning session, the lack of diurnal variations found in FC suggests that we have identified more circadian trait-like differences between the groups. In addition, since LCPs commonly have to get up prior to habitual wake-up time, this study was specifically conducted to investigate these individuals in a “real-world” situation. Dissociating the impact of circadian misalignment and sleep deprivation is often difficult, with protocols such as forced desynchrony and constant routine generally providing the gold standard. However, these protocols have disadvantages in terms of their ability to understand the impact of differences in habitual sleep patterns and circadian phenotype on the brain and behavior. Future work will need to make use of these protocols and to study individuals who are acutely misaligned in order to explore the longer term effect on the brain of chronic misalignment.

### Conclusions

In summary, we find that there are fundamental differences in the intrinsic FC of the DMN between ECPs and LCPs during a typical “societally constrained” working day. rs-FC of the DMN can predict attentional performance measures and subjective sleepiness differences, which are also modulated by time of day. These findings could contribute to the neural basis underlying performance and health differences between ECPs and LCPs in the real world and have implications for future research. Firstly, an individual’s circadian phenotype should be a factor that is taken into account when using fMRI for research and clinical applications, as should habitual sleep status and duration [22]. Secondly, we provide a deeper understanding of the biological basis of individual differences in the DMN that may be associated with negative outcomes in LCPs. Finally, LCPs are impaired during typical socially constrained days, which could result in lower FC and lead to their diminished morning performance and increased daytime sleepiness. This suggests a need to be more conscious about how to manage time on an individual basis in order to maximize productivity and minimize health risks.

## Funding

This work was supported by funding from the Biotechnology and Biological Sciences Research Council (BBSRC, BB/J014532/1) and the Engineering and Physical Sciences Research Council (EPSRC, EP/J002909/1) as well as theFundação de Amparo à Pesquisa do Estado de São Paulo (#2013/07559-3). E.R.F-C. was supported by an Institutional Strategic Support Fund Accelerator Fellowship through the Wellcome Trust (Wellcome, 204846/Z/16/Z).

*Conflict of interest statement.* B.M. and D.J.S. are co-directors of Stockgrand Ltd. The authors declare no other competing financial interests. Non-financial disclosure: none.

## Notes

The work was performed at Centre for Human Brain Health, University of Birmingham, Birmingham, B15 2TT, UK.
